# Thinking about going to the dentist: a Contemplation Ladder to assess dentally-avoidant individuals' readiness to go to a dentist

**DOI:** 10.1186/1472-6831-11-4

**Published:** 2011-01-27

**Authors:** Trilby Coolidge, Erik Skaret, Masahiro Heima, Elissa K Johnson, M Blake Hillstead, Nadia Farjo, Oyvind Asmyhr, Philip Weinstein

**Affiliations:** 1 Dental Public Health Sciences, University of Washington, Seattle WA, USA; 2 Department of Paediatric Dentistry and Behavioural Science, Faculty of Dentistry, University of Oslo, Norway; 3 Department of Pediatric Dentistry, Case Western Reserve University, Cleveland OH, USA; 4 School of Dentistry, University of Washington, Seattle WA, USA; 5 Harvard School of Dental Medicine, Boston MA, USA; 6 Norwegian Defence Medical Services, Oslo, Norway; 7 Pope's Kids Place, Centralia WA, USA

## Abstract

**Background:**

The Transtheoretical Model suggests that individuals vary according to their readiness to change behavior. Previous work in smoking cessation and other health areas suggests that interventions are more successful when they are tailored to an individual's stage of change with regards to the specific behavior. We report on the performance of a single-item measure ("Ladder") to assess the readiness to change dental-avoidant behavior.

**Methods:**

An existing Contemplation Ladder for assessing stage of change in smoking cessation was modified to assess readiness to go to a dentist. The resulting Ladder was administered to samples of English-speaking adolescents (USA), Spanish-speaking adults (USA), and Norwegian military recruits (Norway) in order to assess construct validity. The Ladder was also administered to a sample of English-speaking avoidant adolescents and young adults who were enrolled in an intervention study (USA) in order to assess criterion validity. All participants also had dental examinations, and completed other questionnaires. Correlations, chi square, t tests and one-way ANOVAs were used to assess relationships between variables.

**Results:**

In two samples, participants who do not go to the dentist had significantly more teeth with caries; in a third sample, participants who do not go to the dentist had significantly worse caries. Ladder scores were not significantly related to age, gender, caries, or dental fear. However, Ladder scores were significantly related to statements of intention to visit a dentist in the future and the importance of oral health. In a preliminary finding, Ladder scores at baseline also predicted whether or not the participants decided to go to a dentist in the intervention sample.

**Conclusions:**

The data provide support for the convergent and divergent construct validity of the Ladder, and preliminary support for its criterion validity. The lack of relationship between dental fear and Ladder scores suggests that avoidant individuals may be helped to decide to go to a dentist using interventions which do not explicitly target their fear.

## Background

Health behavior research is increasingly focusing on various theoretical models and their effectiveness and applicability to change negative health behavior in the population. Understanding their strengths and applicability is critical for health care providers who work with preventive health care.

Oral health practitioners, like their peers in other fields, may be surprised when patients do not necessarily change their behaviors when given expert advice to do so. On the face of it, it seems plausible that patients would change their behavior, once they have been educated as to the reasons for the recommendation. While some patients do change upon hearing such recommendations, not all do. The Transtheoretical Model (TTM) posits that behavior change is multi-phasic and can be characterized as occurring along a continuum. With regards to any particular behavior, a patient may be characterized as being in the initial stage, or Precontemplation, in which the individual is not thinking about changing. Another patient may be thinking about making the change, but has not yet decided to do so (likely due to ambivalence about making the change), in the Contemplation stage. Finally, a third may have weighed the situation and decided to make the change, in the Action stage [1].

This continuum was first described to characterize the various methods used by individuals who had quit smoking on their own, without undergoing any specific intervention for that purpose [2]. Addiction counselors have increasingly used the model to tailor their interventions to the stage at which the patient is currently at. For example, a patient who is referred to a drug and alcohol counselor by his/her physician may present anywhere on the continuum, from denying that he/she has a problem (Precontemplation), through acknowledging that he/she has thoughts about quitting but still finds the problematic behavior rewarding in some way and/or worries about possible negative consequences of quitting (Contemplation), to expressing a desire to quit but still grappling with potential stumbling blocks (Action). The TTM further posits that individuals will respond to different intervention strategies according to the stage that they are currently in. For example, an individual in the Precontemplative stage who is told "You should change your behavior because it is unhealthy" is likely to respond with disinterest or even denial that he/she has any need to change. The goals of the counselor using the TTM model are to ascertain what stage the patient is in, and then tailor the intervention to that stage with the ultimate aim of encouraging the patient's movement to the next stage.

Such tailored interventions have been successful with a number of addictive behaviors ranging from drug and alcohol use to pathological gambling [3,4]. This approach has also been useful in helping people make "lifestyle" changes, such as increasing exercise, modifying diet, changing high-risk sexual behaviors, adhering to medication regimens, participating in mental health prevention programs, and the like [5-7].

The preceding examples share the feature that the behavior in question occurs often, perhaps even daily or multiple times a day. The tailored approach has also been effective with less frequent behaviors, such as motivating avoidant women to obtain medical screenings for breast and cervical cancer [8,9], and motivating infrequent blood donors to donate on a regular basis [10].

From an oral health perspective, it would be desirable to influence dentally-avoidant individuals to decide to seek out dental care. According to the TTM model, dentally-avoidant individuals might be not thinking at all about going to a dentist (i.e., are in the Precontemplation stage), or they may be considering contacting a dentist but haven't decided, due to unresolved ambivalence about going (i.e., are in the Contemplation stage), or they may have decided to contact a dentist (i.e., are in the Action stage). Depending on the stage, tailored interventions could include "I respect you to make your own decision. If it's OK with you, I'd like to share what we are learning about the value of dental visits" (Precontemplation), "In your opinion, what are the benefits of seeing a dentist, and what are the downsides? How do they fit together, would you say?" (Contemplation), or "OK, so you've decided to see a dentist, but you're still a bit worried. Let's talk about how you could find a dentist who would be sensitive to your concerns" (Action) [1]. According to this model, it is important to be able to assess the stage of change a dentally-avoidant individual is in and use this information to select stage-appropriate interventions.

Stage of change measures have been developed for a number of behaviors. One common measure, the University of Rhode Island Change Assessment (URICA), is a 32-item questionnaire assessing an individual's level on each of four subscales: Precontemplation, Contemplation, Action, and Maintenance (i.e., maintaining the behavior change) [11]. Items are written in such a way that they can be applied to any problematic behavior; for example, one item on the Contemplation subscale reads "I have a problem and I really think I should work on it." While frequently used, many authors report that the subscales are intercorrelated, reducing its ability to assign individuals to a particular stage [12]. An additional drawback to this measure is its length.

One promising alternative to the URICA and other lengthy stages of change measures is the "Contemplation Ladder", a single-item measure consisting of a drawing of a ladder whose rungs are numbered from 0 to 10. Some of the rungs have statements assigned to them, and the individual is asked to select which rung (number) best represents his/her thinking and/or actions at the present time about the potential behavior change, using the statements as guides. For example, the original ladder, developed for assessing readiness to quit smoking, states "Each rung on this ladder represents where various smokers are in their thinking about quitting. Circle the number that indicates where you are now", while the statements assigned to specific rungs include "No thought of quitting" (at the bottom of the ladder, assigned to 0), and "Taking action to quit" (at the top of the ladder, assigned to 10), as well as others for the intermediate rungs [13]. The Contemplation Ladder for smoking cessation has been found to be valid in assigning the stage of change as well as in predicting future smoking cessation [12-16].

Modifications of the smoking cessation ladder have been validated for assessing readiness to make other health behavior changes, such as increasing physical exercise, decreasing anorexic behaviors, reducing alcohol use, and reducing marijuana use, and successfully used in interventions targeting these behaviors [17-20]. Modifications of the ladder have also been used in reducing problematic gambling [21], understanding the factors in physicians' readiness to recommend colonoscopy to patients [22], and assessing readiness to seek employment in a sample of under- and unemployed welfare recipients [23]. In addition to studies with adults, the original and modified ladders have been found to be valid for adolescent smokers and marijuana users [15,20,24].

Establishing the validity of a readiness to change measure would be important in intervention studies focused on other health behaviors which are based on the TTM model. We are particularly interested in intervening with dentally-avoidant individuals. Because of its brevity, good criterion validity, and acceptance by both adolescents and adults, we believe that a version of the contemplation ladder might be useful to predict readiness to go to a dentist in individuals who are dentally avoidant.

In this paper, we report on data obtained from four samples (each part of a larger study) which reflect the performance of the Thinking About Going to the Dentist Contemplation Ladder (for brevity's sake, hereafter referred to as the Ladder). The overall aims were to explore the construct and criterion validity of this version of the Ladder. In particular, we hypothesized that participants who selected higher scores on the Ladder would also give stronger endorsements to statements enquiring about future intentions to go to a dentist and attitude towards their oral health, as well as fewer negative beliefs about dentists, as examples of convergent construct validity. On the other hand, we hypothesized that age and gender might not be related to Ladder scores. Also, given our clinical experience treating formerly-avoidant individuals who decide to seek dental care, and who present with wide variations in carious status as well as dental fear, we hypothesized that these two variables might be independent of Ladder scores. Thus, these four variables (age, gender, carious status, dental fear) were assessed to examine the divergent construct validity of the Ladder. Finally, we predicted that higher Ladder scores would be found in participants who subsequently reported going to a dentist, as a measure of the Ladder's criterion validity.

## Methods

The University of Washington IRB approved the studies carried out in Washington State (Samples 1, 2, and 4), while the Regional Committees for Research Ethics and Norwegian Social Science Data Services approved the Norwegian study (Sample 3).

### Materials

**Figure 1:**
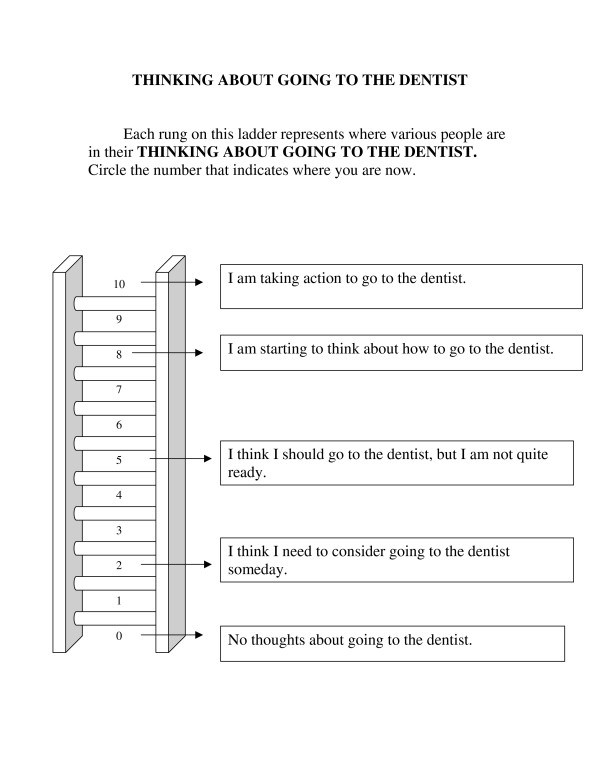
**Thinking About Going to the Dentist Contemplation Ladder**.

The Ladder was based on the original smoking cessation ladder developed by Biener and Abrams [13] by substituting wording related to going to the dentist for the original wording, which referred to quitting smoking. For example, the original wording for the middle rung is "Think I should quit but not quite ready", while the revised wording for our studies is "I think I should go to the dentist, but I am not quite ready". The English version of the Ladder is shown in Figure 1. The Spanish and Norwegian versions of the Ladder were made by translating and back-translating the English wording into the two languages by two independent bilingual individuals for each version. The wording for the Spanish and Norwegian versions is shown in additional file 1 (Spanish and Norwegian versions of the Ladder). For scoring purposes, we reassigned the values of the rungs to range from 1 (lowest rung) to 11 (highest rung).

Other questionnaires used in the samples included the Spanish Modified Dental Anxiety Scale (Spanish MDAS) [25], the Norwegian Dental Anxiety Scale (Norwegian DAS) [26], the Dental Neglect Scale (DNS) [27], the Revised Dental Beliefs Survey (R-DBS) [28], and other items assessing current, prior, and/or future dental attendance. Table 1 provides a summary of the questionnaires used in each sample, as well as a summary of each sample's overall study design.

**Table 1:** Study Designs of the Four Samples

Sample	1	2	3	4
**Design**	**Cross-sectional**	**Cross-sectional**	**Cross-sectional**	**Longitudinal**

**Selection Criterion**	**Does not go to dentist**	**Does not go to dentist**	**Does not go to dentist**	**Presence of severe caries**

Questionnaires:				
Ladder	Yes	Yes	Yes	Yes
Dental Neglect Scale	Yes			
Modified Dental Anxiety Scale		Yes		
Dental Anxiety Scale			Yes	
Revised Dental Beliefs Survey				Yes
Question About Future Dental Visits		Yes	Yes	

Dental Examination: Field vs. Traditional	Field	Field	Traditional	Field

Dental Disease:				
Number of Carious Teeth	Yes	Yes	Yes	No^1^

Severity of Caries	Yes	Yes	Yes	No^1^

^1^Severe caries was the criterion for inclusion in this sample.

### Oral Examinations

In addition to completing questionnaires, all participants underwent one of two oral examinations. A summary of the type of examination used for each sample is shown in Table 1. In the three Washington State samples (Samples 1, 2 and 4), data were collected in the field and the oral examination was brief (using light and mirror only) according to WHO criteria [29]. The participants in Sample 4 had been screened previously by the WHO protocol and were eligible for this study if they were found to have extensive visibly untreated caries [30], while the participants in Samples 1 and 2 underwent the WHO screening as part of this study. The WHO protocol was selected because it had been successfully used by members of the study team in one of the settings in the past [31], was rapid enough to fit the time constraints of the current settings (none of which were dental offices or permitted radiographs), and was easily learned by our dental personnel. Instruction, discussion and demonstration with photographs was followed by calibration on live individuals until examiners reached a minimum consensus (kappa = .85 or higher) with the senior calibrating dentist; overall, kappas ranged from .85 to 1.0 [30].

The Norwegian sample (Sample 3) involved military recruits undergoing physical and dental assessments, and radiographs were part of the oral examination. Therefore, a protocol suitable for typical dental examination was chosen (rather than the field orientation of the WHO protocol used in the other three samples). The protocol and diagnostic system of Amarante et al. [32] was selected, in part because the researcher in Amarante's group who had developed the system was available for the calibration of the dental personnel in Norway. Instruction in the system and discussion was followed by calibration with live individuals. After initial calibration, the dental personnel re-rated a subset of radiographs after an interval of 60 days. Over this interval, kappas ranged from .60 to .80.

### Participants and Procedures

Table 2 presents a summary of the participant characteristics in each of the four samples.

**Table 2:** Participant Characteristics in the Four Samples

Sample	1	2	3	4
**General Characteristics**	**Adolescents in Youth Groups**	**Spanish-speaking adults**	**Norwegian military recruits**	**Rural adolescents/young adults with severe caries**

Total Number of Participants	126	162	1984	24

Number of Avoidant Participants	33	77	237	24

Language	English	Spanish	Norwegian	English

Age	12 - 18	18 - 64	19 - 22	13 - 28

Payment	Yes	Yes	No	Yes

### Sample 1: Adolescents attending youth clubs

One hundred twenty six English-speaking adolescents aged 12 - 18 who were members of youth groups in the Seattle-Tacoma (Washington State) area participated in a study whose primary purpose was to assess the psychometric properties of the DNS in adolescents, and were paid for their time [33]. After completing a questionnaire, adolescents underwent a brief oral examination (light and mirror only) according to WHO criteria [29]. As reported by Coolidge et al. [33], adolescents who do not go to the dentist scored significantly worse (more neglectful) on the two DNS items related to visiting a dentist, compared with their peers who do go to a dentist, while there were no differences on the three items related to oral self care behaviors. Of note, there was also no significant group difference on the single attitudinal item in this scale: "I consider my dental health to be important". The adolescents who stated that they did not go to a dentist were asked to complete the Ladder, and we report on these data here.

### Sample 2: Spanish-speaking adults

One hundred sixty two Spanish-speaking adults attending either Spanish-language church services or an Hispanic festival in Washington State were recruited to participate in a larger study designed to explore the relationships between dental attitudes, dental attendance behavior, and oral health in Spanish-speakers. Participants completed questionnaires containing the Spanish MDAS and other questionnaires not reported here, and an item asking if they currently go to a dentist. Participants who answered "no" were then asked to complete the Spanish Ladder. They were also asked about future dental attendance with an item reading "Are you seriously considering going to the dentist in the next year?" which was answered yes or no. Following the questionnaire administration, participants were invited to undergo a brief oral examination, using the same procedures as in Sample 1 (see [34] for details). Participants were paid for their time.

### Sample 3: Norwegian adults

As part of a larger health study, 1984 military recruits completed the Norwegian DAS and answered questions about prior dental attendance patterns. They were also asked about future dental attendance with a single item which read: "How likely is it that you will go to the dentist during the next 5 years?" The item was answered on a 5-point scale, ranging from "very likely" to "very unlikely". Since all recruits received a dental examination at the same time (after having completed the questionnaire), and therefore were seeing a dentist at the time of the questionnaire administration, the wording of the instructions for the Norwegian Ladder was adjusted to reflect this (see additional data file #1: Spanish and Norwegian versions of the Ladder). To provide an additional check on the performance of this Ladder with the adjusted wording, it was administered to all participants so that the results of avoidant and non-avoidant participants could be compared. The recruits then underwent a dental examination including radiographs; caries diagnosis was determined according to the criteria outlined in Amarante et al. [32]. The recruits were not paid.

### Sample 4: Rural adolescents and young adults

Forty seven English-speaking adolescents and young adults in a rural county in Washington state who had extensive visibly untreated caries, considered to be indicative of failure to visit a dentist for a year or longer [35,36], were recruited into a pilot study to explore counseling interventions to encourage them to decide to seek out dental care (see [30] for further information about the dental screening and recruitment procedures). Half (24) of them were randomly assigned to an intervention stressing the importance of visiting a dentist, while the other half (23) were randomly assigned to an alternate intervention. Due to procedural mistakes, the counselor for the second condition (alternate intervention) misplaced all but one participant's study records, and therefore data from these 23 participants are not included here.

The remaining 24 participants completed a questionnaire containing the Ladder, the R-DBS, demographic items, and other items not reported here. Following this, they received the counseling intervention, delivered according to a script in one in-person session. All participants received the same intervention in this condition, regardless of their Ladder score (stage of change). Sessions were audiotaped and transcribed to ensure treatment fidelity. The counselor made three follow-up contacts, also following scripts, at one-month intervals. At each follow-up contact, the participant was asked if he/she had decided to see a dentist. Participants also had the option of contacting the counselor between scheduled contacts, if he/she had decided to see a dentist and wanted her help to find a dentist. Participants were coded as "successes" if they stated that they had decided to see a dentist, or had told the counselor that they wanted to see a dentist, at any time during the follow-up period. Participants were paid for their time.

### Analyses

Data were entered in Excel spreadsheets using double entry for accuracy. If a participant gave two answers to a questionnaire item, the mean value was substituted and entered into the corresponding data base. Analyses were carried out with SPSS Versions 14.0 (Samples 1, 2 and 4) and 16.0 (Sample 3). Only complete questionnaires were used in analyses which included questionnaire sums. In addition to frequencies, correlations, chi square, and t tests were used to assess the relationships between variables, depending on whether the variables were continuous or categorical. One-way ANOVAs were computed to examine possible differences between Ladder scores and caries severity. Because we predicted that higher Ladder scores would be positively related to the scores obtained on the items asking about future intentions to go to a dentist and the importance of one's health, as well as inversely related to the number of negative beliefs about dentists (tests of convergent validity), the analyses between these variables were examined with one-tailed tests. Similarly, because we predicted that higher Ladder scores would be related to the decision to seek dental care (criterion validity), this analysis was designed to be one-tailed. On the other hand, because we predicted that the Ladder scores might be independent of age, gender, caries status, and dental fear (tests of divergent validity), the analyses between these variables were two-tailed.

## Results

Tables 3 and 4 provide summaries of the Ladder scores found in each sample, and the relationships between Ladder scores and the other variables measured to assess construct and criterion validities.

**Table 3:** Ladder Scores in the Four Samples

Ladder Scores:		Mean	SD	Range
Sample 1		7.93	3.23	1-11

Sample 2		7.67	2.91	1-11

Sample 3:				
	Avoidant Participants	7.45	3.02	1-11
	Non-Avoidant Participants	8.71	2.83	1-11

Sample 4		9.48	2.04	4-11

**Table 4:** Relationships Between Ladder Score and Other Variables Assessing Convergent and Divergent Validities and Criterion Validity

Sample	1	2	3	4
Convergent Construct Validity:				
I consider my dental health to be important (DNS)	Rho = 0.51**			
I plan to go to the dentist		t = 2.236*	Rho = 0.32*** t = 5.082***	
Beliefs about dentist (R-DBS)				Negative^1^

Divergent Construct Validity:				
Age	NS	NS	NS	NS
Gender	NS	NS	NS	NS
Number of Carious Teeth	NS	NS	NS	NA^2^
Severity of Caries	NS	NS	NS	NA^2^
Dental Fear (MDAS Continuous)		NS		
Dental Fear (MDAS High vs. Low)		NS		
Dental Fear (DAS Continuous)			NS	
Dental Fear (DAS High vs. Low)			Trend	

Criterion Validity:				
Decides to go to dentist				Positive^3^

* p < 0.05

** p < 0.01

*** p < 0.001

NS = Not statistically significant

NA = Not applicable

^1 ^Those who decided to see a dentist had higher (more negative) R-DBS scores, but the difference was not tested due to the low sample size.

^2 ^Severe caries was the criterion for inclusion in this sample.

^3 ^Those who decided to see a dentist had higher Ladder scores, but the difference was not tested due to the low sample size.

### Sample 1: Adolescents attending youth clubs

Among the 126 participants, 33 stated that they did not currently go to a dentist. Their mean age was 14.97 years (SD = 2.21, range = 12 - 18), and 58% were males. The adolescents who do not go to a dentist had a mean of 1.45 teeth with visibly untreated caries (SD = 2.22, range 0 - 9), compared with a mean of 0.60 teeth with visibly untreated caries observed in the adolescents who do go to a dentist (SD = 1.18, range 0 - 5). This difference was significant (t = 2.106, df = 39.332, p = 0.042).

The mean Ladder score of the adolescents who do not go to a dentist was 7.93 (SD = 3.23, range 1 - 11). Ladder scores were not related to age, gender, number of teeth with visibly untreated caries, or severity of visible caries. However, adolescents who more strongly endorsed the DNS item regarding their attitude towards their own oral health ("I consider my dental health to be important") had significantly higher Ladder scores (Spearman's rho = 0.51, p = 0.003).

### Sample 2: Spanish-speaking adults

Nearly half (77) of the adults stated that they do not go to a dentist. The mean age of those who do not go to a dentist was 37.22 years (SD = 11.42, range = 18 - 64), and 59.2% were female. They had a mean number of 1.99 teeth with visibly untreated caries (SD = 2.56, range 0 - 14), compared with a mean of 1.18 teeth with visibly untreated caries (SD = 1.53, range 0 - 7) in those who do go to a dentist. This difference was significant (t = 2.363, df = 151, p = 0.019).

Most (72) of the participants who do not go to a dentist completed the Ladder. Their mean Ladder score was 7.67 (SD = 2.91, range 1 - 11). Ladder scores were not related to age, gender, number of teeth with visibly untreated caries, or severity of visible caries. Ladder scores were also not related to the MDAS, whether the MDAS was measured as a continuous variable or as a categorical one (high fear vs. low fear; MDAS > = 19 represents high fear). However, Ladder scores were significantly higher for those who stated that they were seriously considering going to a dentist, compared to those who said that they were not (mean value for those seriously considering going to a dentist = 7.89, SD = 2.77; mean value for those not seriously considering going to a dentist = 5.00, SD = 3.08; t = 2.236, df = 68, p = 0.015).

### Sample 3: Norwegian adults

The mean age of the participants in this sample was 20.7 years (SD = 0.90, range 19 - 22), and 96.9% were male. Of these, 237 (12.1%) had not been to the dentist for at least two years, and were considered to be avoidant for this study. A total of 99.2% of the avoidant group were male, and the mean age of those in the avoidant group (mean = 21.2 years, SD = 0.81, range = 19 - 22) was significantly higher than those who were not avoidant (mean age = 20.6 years, SD = 0.87, range = 19 - 22; t = 10.59, df = 1956, p < 0.05). Those in the avoidant group had significantly more carious teeth than did the recruits who had been to a dentist in the last two years (mean = 5.52, SD = 4.58 vs. mean = 4.47, SD = 3.90; t = 5.46, df = 1902, p < 0.001). The avoidant group had a mean number of 0.28 teeth with severe caries (D5 [32]) (SD = 0.90, range 0 - 7), compared with a mean of 0.06 teeth (SD = 0.51, range 0 - 15) in those who had been to the dentist in the past two years. This difference was significantly different (t = 5.459, df = 1902, p < 0.001).

**Figure 2:**
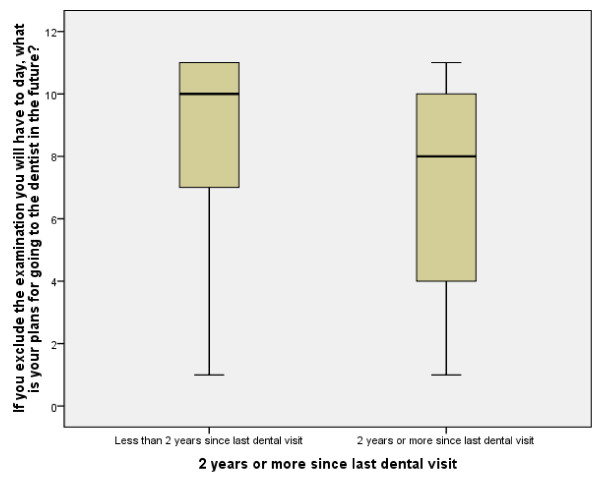
**Ladder scores for non-avoidant and avoidant participants in Sample 3**.

The mean Ladder score of the avoidant participants was 7.45 (SD = 3.02, range = 1 - 11), while the mean Ladder score for the non-avoidant participants was 8.71 (SD = 2.83, range = 1 - 11). The mean values were significantly different (t = 6.35, df = 1929, p < 0.001). The mean Ladder values for the avoidant and non-avoidant participants are also presented in Figure 2.

There was no relationship between age or gender and Ladder scores for either the avoidant or non-avoidant participants. There were also no relationships between Ladder scores and either the number of decayed teeth or the number of teeth with severe caries lesions (D5 [32]). The mean value on the Norwegian DAS for the avoidant participants was 8.71 (SD = 4.07, range 4 - 20). For the non-avoidant participants, the mean score on this measure was 7.34 (SD = 2.94, range 4 - 20). This difference was significant (t = 6.34, df = 1936, p < 0.001).

There was no significant relationship between dental fear and Ladder scores in the avoidant participants when this was assessed using DAS scores as a continuous variable. Participants with high dental fear (DAS > = 13) had a mean Ladder score of 6.72 (SD = 2.77), while those with low dental fear had a mean Ladder score of 7.63 (SD = 3.08); this difference showed a trend towards statistical significance (t = 1.840, df = 229, p = 0.067).

Avoidant participants who stated that they were more likely to visit a dentist in the future had significantly higher Ladder scores, (Spearman's rho = 0.32, p < 0.001). When future likelihood was dichotomized into likely (likely or very likely) vs. unlikely (unlikely or very unlikely), those who stated that they were likely to go to the dentist in the next 5 years had significantly higher scores on the Ladder (mean = 8.94, SD = 2.46), compared with participants who reported that it is not likely that they will go to the dentist in the next 5 years (mean = 6.34, SD = 3.13; t = 5.082, df = 136, p < 0.001).

### Sample 4: Rural adolescents and young adults

The mean age of the 24 participants was 19.48 years (SD = 4.71, range 13 - 28), and 65.2% were female. Their mean Ladder score was 9.48 (SD = 2.04, range 4 - 11). Three participants were lost to follow-up (one moved out of state, one lost her housing and was reported to be living in her car, and the third did not respond to numerous phone calls and letters). The remaining 21 participants had a mean age of 19.40 (SD = 4.86, range 13 - 28), and 65% of them were female. The mean Ladder score for the remaining participants was 9.88 (SD = 1.58, range 5 - 11).

By the one-month follow-up contact, 13 participants stated that they had decided to see a dentist. By the two-month contact, an additional 6 stated that they had decided to go to a dentist. No additional participants stated that they wanted to see a dentist by the three-month follow-up. Thus, 19 decided to see a dentist, while 2 did not. The mean Ladder score for those who decided to see a dentist was 10.29 (SD = 0.90, range = 9 - 11), compared with a mean score of 6.00 (SD = 1.41, range = 5 - 7) for those who did not. Because of the small number of participants who did not decide to see a dentist, no statistical test of the difference between the means was computed.

Neither age nor gender was related to whether or not a participant decided to see a dentist. The participants who decided to see a dentist had more negative beliefs about the dentist (mean R-DBS sum score of 61.38 [SD = 25.90, range = 28 - 121] for those who decided to see a dentist, compared with mean R-DBS sum score of 56.00 [SD = 22.63, range = 40 - 72] for those who did not).

## Discussion

Data from the first three samples provide evidence for the convergent construct validity of the Ladder. Specifically, adolescents in the first sample who do not go to a dentist but more strongly endorsed the DNS item "I consider my dental health to be important" had significantly higher Ladder scores, indicating congruence between a positive attitude towards dental health and a readiness to act consistently with this by going to a dentist. In the Norwegian sample, the perceived likelihood of seeing a dentist in the future was significantly related to Ladder scores in military recruits who had not been to a dentist for two or more years, while Spanish-speaking adults who do not currently go to a dentist but state that they are seriously considering going in the next year endorsed significantly higher Ladder scores than those who are not considering going. Taken together, the Norwegian and Spanish results suggest that the Ladder and questions about intentions to go to a dentist in the future are assessing similar constructs.

The data also provide evidence for the divergent construct validity of the measure, in that there were no significant relationships between age or gender and Ladder scores; more importantly, there was no significant relationship between dental fear and readiness to go to a dentist in the Spanish sample, and only a trend in the larger Norwegian sample. While dental fear is higher in general in those who are avoidant [37-39], our data suggest that the decision that formerly-avoidant individuals make to go to a dentist may be independent of their fear. That is, fearful individuals may decide to seek out dental care. This is consistent with a considerable body of previously-published findings, both in clinical and population studies [28,40,41]. This is also similar to some data with cigarette smokers, in that the level of nicotine dependence may be weakly related, or even not related, to the stage of change that the smoker is in with regards to nicotine cessation [13,15]. That is, while fear -- or nicotine dependence -- may be important variables in understanding current avoidant (or smoking) behavior, the desire to change may not be directly related to them. This finding also suggests that there may be useful methods to encourage dentally-avoidant individuals to seek out dental care without necessarily directly targeting their fear.

The participants in the English-language adolescent and Spanish-language adult samples were self-selected from these two convenience samples, and therefore our results cannot necessarily be extrapolated to other adolescents or Spanish-speaking adults without further research. By contrast, the Norwegian sample was a random sample of all military recruits in their cohort. All children in Norway receive free dental care until the age of 18, although some adolescents avoid receiving this free care [42]. Following this, young adults aged 19 - 20 are entitled to greatly-reduced fees for dental services. All young Norwegian men (and, recently, some women) aged 17 - 18 take part in a compulsory general health screening in the Norwegian Defence Medical Services, and those who are found to be healthy are recalled to a second examination at approximately age 20 - 21. Our data were collected at this second examination. It is of interest to note that the proportion of our Norwegian sample that had not been to the dentist in two or more years (12.1%) is similar to that seen in the entire adult population in Norway (11%) [43]. Further, the Norwegian Defence has conducted a stratified random sample of all young Norwegian adults of this age (i.e., including those who would have been screened out at the initial examination due to poor health), matched for the age and gender of those who were included in the second screening, and preliminary data analyses reveal that there are no significant differences in attitudes towards dental attendance and dental attendance habits between the recruits and the same age and gender young adults in general (personal correspondence, E. Skaret, October 25, 2010). One weakness of our Norwegian sample is that it consists of a very narrowly aged, primarily male, cohort. On the other hand, the sample has uniformly experienced a lifetime of free or nearly free dental care, and in several ways (including avoidance) appears to be similar to their peers and to the Norwegian population as a whole. These strengths add credence to the construct validity results reported for this sample.

Although the sample size is too small to be definitive, in our fourth sample (rural adolescents and young adults) we did not find that participants with more negative beliefs about the dentist were less likely to decide to seek dental care. Interestingly, we found that those with more negative beliefs were actually more likely to decide to see a dentist. This was somewhat surprising, given that others have found that dental avoiders are more likely to have higher scores on the original Dental Beliefs Survey [39]. If replicated in larger samples, the apparent lack of relationship between the R-DBS and decision to see a dentist in dental avoiders indicates that the Ladder is tapping a construct which is independent of negative perceptions of the dentist, and thus may also indicate that there may be ways to encourage avoidant individuals to seek dental care without directly challenging their negative beliefs.

This sample (rural adolescents and young adults) was also designed to assess the criterion validity of the Ladder. Our results indicate that baseline Ladder scores appear to be higher for individuals who decided to seek dental care within 3 months. Compared with the three other samples described here, wherein participants' Ladder scores expressed the full possible range (i.e., 1 to 11), the avoidant adolescents and young adults had a narrower range of Ladder scores, which was also reflected in their higher mean scores. Ordinarily, the narrower range would make it harder to find differences between groups. Thus, despite the small sample size, the difference in means implies that the Ladder may have good criterion validity. Nevertheless, this finding would be best considered a preliminary one at this time, and needs to be replicated in a larger sample.

In this sample (rural adolescents and young adults), all participants received a counseling intervention after completing a baseline questionnaire which included the Ladder. The potential impact of the counseling intervention on the decision to see a dentist is likely to be largely mitigated by the fact that all individuals received an identical intervention. In addition, there is evidence that baseline Ladder scores mediate the effects of interventions [19], which means that both baseline readiness and responses to interventions are important in predicting behavior change. A stronger test of the relationships between baseline Ladder scores and active intervention on behavior change would require a controlled study. As noted above, by mistake the vast majority of the data from the alternate counseling condition were lost. In our design, the lost data were from a condition which incorporated baseline Ladder scores into tailored interventions, consistent with the TTM model, while the data we report on here were meant to be from a control (non-tailored) intervention. While the existing data are easier to interpret, in that all participants received identical interventions, unfortunately our final sample size was lower than desired, and we are unable to look for relationships between the Ladder, varieties of (tailored) intervention, and behavioral change. We hope that future controlled studies can examine these relationships.

As noted previously, one of the attractive features of the single-item ladders is their brevity, especially compared with the URICA and other lengthy stage of change measures. Ladders also have been validated for a variety of target behaviors, indicating that the potential use for this kind of measure is broad. It is interesting to speculate on the ways in which the ladders differ from simple 10-point scales ("On a scale of 1 to 10, where 1 means ... and 10 means ..., how likely are you to...?"). First, the ladders include several declarative, first-person sentences, rather than just the anchors for the lowest and highest numbers, which may impact how individuals evaluate the intermediate numbers on the measure. Secondly, the ladders include a graphic, which may make them more appealing than simple verbal descriptions. Third, perhaps the graphic of a ladder implicitly contains the message that one could "climb up" towards action, with regards to the behavior or problem under consideration. To our knowledge, none of these features of ladders have been tested by comparing ladders to the simple 10-point scales. Future research might examine these hypotheses.

## Conclusions

In sum, data from our studies indicate that the Ladder has good construct validity, and at least on a preliminary basis has good criterion validity, as well. Further studies with other samples would be needed to provide more evidence for this measure's validity in assessing the readiness to go to a dentist in individuals who are dentally-avoidant.

## Competing interests

The authors declare that they have no competing interests.

## Authors' contributions

TC conceived of the idea of developing a contemplation ladder for assessing readiness to change dental behavior, designed the study reported in Sample 1, oversaw the translation of the Ladder into Spanish, oversaw the data collection reported in Samples 1, 2, and 4, collected data in Samples 1 and 2, conducted the data analyses for Samples 1, 2, and 4, and wrote the manuscript. ES, in consultation with TC, contributed to the questionnaire design used in the larger Norwegian study and oversaw the translation of the Ladder into Norwegian. PW, with assistance from TC, designed the protocol for Sample 4. MH trained the dental personnel for the oral examinations in Samples 1, 2 and 4. EKJ collected and entered data from Sample 1, and MBH and NF collected and entered data from Sample 2. OA was responsible for, and oversaw the data collection for Sample 3, and ES and OA entered the data and conducted the data analyses for Sample 3. All authors have read and approved the final manuscript.

## Pre-publication history

The pre-publication history for this paper can be accessed here:

http://www.biomedcentral.com/1472-6831/11/4/prepub

## Supplementary Material

Additional file 1 **Spanish and Norwegian versions of the Ladder**. Click here for file
