# Supplementary material for: Thinking about going to the dentist: a Contemplation Ladder to assess dentally-avoidant individuals' readiness to go to a dentist
Source: BMC Oral Health. 2011 Jan 27;11:4. doi: 10.1186/1472-6831-11-4 (PMC3045398; doi:10.1186/1472-6831-11-4)
Supplement: Additional file 1 — Spanish and Norwegian versions of the Ladder. [file 1472-6831-11-4-S1.DOC]

Additional file 1: Spanish and Norwegian versions of the Ladder

Spanish version of the Ladder:

Cada escalón in esta escalera representa el lugar en el que varias personas están situadas con respecto al pensamiento acera de ir al dentista. Coloca un círculo alrededor del número que indica donde estas tu ahora. [Each rung on this ladder represents where various people are in their thinking about going to the dentist. Circle the number that indicates where you are now.]

Rung 10: Estoy tomando acciones par ir al dentista.

Rung 8: Estoy empezando a pensar sobre cómo ir al dentista.

Rung 5: Pienso que debería al dentista, pero aún no estoy muy preparado.

Rung 2: Pienso que debería considerar la posibilidad de ir al dentista algún día.

Rung 0: No he pensado ir al dentista.

Norwegian version of the Ladder:

Hvis du ser bort fra den undersøkelsen du får i forbindelse med dette inntaket - Hva slags planer har du når det gjelder å gå til tannlegen for fremtiden? Sett ett kryss ved det tallet i stigen som indikerer hvor du er for tiden [If you exclude the examination you will have today, what are your plans for going to the dentist in the future? Circle the number that indicates where you generally are ] **.**

Rung 10: Jeg er klar for å gå til tannlegen

Rung 8: Jeg begynner å tenke på hvordan jeg skal komme meg til tannlegen

Rung 5: Jeg tror jeg bør gå til tannlegen, men jeg er ikke helt klar for det

Rung 2: Jeg tror jeg bør vurdere å komme meg til tannlegen en eller annen gang

Rung 0: Jeg har ingen planer om å gå til tannlegen
